# Exploring potential factors influencing perinatal mental health in Kajiado County, Kenya: a qualitative study with mothers and healthcare workers

**DOI:** 10.1186/s12889-026-28282-4

**Published:** 2026-06-23

**Authors:** Caren Kamau, Sebastian Gabrielsson, Francis W. Makokha, Timothy Ntinina, Jane Karimi, Lydia Munteiyan, Kristian Ekman, Louise Öhlund, Ursula Werneke

**Affiliations:** 1 County Department of Health, Kajiado, Kajiado County Kenya; 2 Directorate of Research and Development, Mount Kenya University, Thika, Kenya; 3 Department of Health, Education and Technology, Luleå University of Technology, Luleå, Sweden; 4 Department of Psychology, Mount Kenya University, Thika, Kenya; 5 Region Norrbotten, Luleå, Sweden; 6 Department of Clinical Sciences, Sunderby Research Unit, Division of Psychiatry, Umeå University, Umeå, Sweden

**Keywords:** Depression, perinatal, Mental health, Rural health, Health care, Risk factors, Mothers, Kenya

## Abstract

**Background:**

Perinatal depression affects up to 30% of women in Kenya, yet many contributing factors remain poorly understood. We explored mental health problems, social needs, and associated risk factors among women ante- and postnatally in Kajiado County, southern Kenya, from the perspectives of women and health-care workers (HCWs).

**Methods:**

We conducted a qualitative study based on dual-perspective focus group interviews with mothers (ante- and postnatal) and HCWs in five health-care facilities (two urban and three rural) across Kajiado County (5–11 March 2025). Interviews were recorded, transcribed verbatim, translated into English when needed, and analysed using qualitative content analysis with an explorative and inductive approach. The text was divided into meaning units and manually coded in Atlas.ti (https://atlasti.com). Themes, subthemes and categories were articulated following a stepwise, iterative and reflexive process of abstraction and interpretation.

**Results:**

Across five sites, 51 mothers and 39 HCWs participated. Three themes were developed relating to stress, distress and affecting mental wellbeing, (a) Women’s autonomy and self-determination – mothers lacked control over resources or decision-making, while shouldering most household responsibilities, and HCWs highlighted the vulnerability of girls subjected to early marriage and female genital mutilation, often unprepared for motherhood, (b) Responsive maternal and mental health care – distance, costs, and low expectations limited access, while women feared traumatic births, miscarriage, and caesarean sections, and (c) Community knowledge and acceptance of mental health problems – mental health problems were often seen as irrationality or spiritual possession, delaying care. Stigma was particularly associated with caesarean sections, mental health problems, and HIV.

**Conclusions:**

Our findings describe how maternal mental health is closely intertwined with gender norms and prevailing perceptions of mental illness including stigma. Expanding maternal mental health services may be important but it is unlikely to be sufficient in isolation. Sustainable change may depend on the promotion of women’s rights, increased mental health literacy at the community level, and the engagement of men. In addition, fear of traumatic birth and stigma associated with CS may need to be addressed not only at the individual level but also within the broader community context.

**Supplementary Information:**

The online version contains supplementary material available at 10.1186/s12889-026-28282-4.

## Introduction

Perinatal mental health problems refer to mental health conditions occurring from pregnancy (antenatal period) to the first year after childbirth (postnatal period). Perinatal depression affects about 20% of women globally and about 25% in low-income and middle-income countries [1, 2]. Generalised anxiety and post-traumatic stress disorder (PTSD) may affect 30% of mothers perinatally [3]. In contrast, postpartum psychosis is rare with global incidence estimates ranging from 0.89 to 2.26 per 1000 births [4]. For Kenya, recent studies suggest a prevalence of around 30% for antenatal depression [5, 6] and 15–27% for postnatal depression [7, 8]. Most studies use localised samples, limiting generalisability. To date, no study has examined perinatal mental health in Kajiado county – a mixed urban-rural county in southern Kenya.

Multiple factors contribute to perinatal mental health problems, including poor nutrition, food insecurity, poverty, intimate partner violence (IPV), lack of social support, and early or unintended pregnancy. Human immune deficiency virus infections (HIV) and the aftermaths of the corona virus-19 disease (COVID-19) pandemic further increase vulnerability [9–11], as do pastoralist livelihoods [12].

Risk factors for perinatal mental health problems vary by setting and population studied. For example, in informal settlements in Nairobi, low education, body image dissatisfaction, family conflicts, and unplanned pregnancy have been associated with postnatal depression [8]. In Western Kenya, IPV, low social support and partner’s HIV-positive status have been linked to moderate to severe depressive symptoms among HIV-negative women [10]. Co-occurring HIV, food and water insecurity have also been shown to increase the risk of depressive symptoms [13].

Qualitative research among women with HIV has highlighted financial insecurity and lack of partner support as key stressors [14]. In pastoralist settings, unequal household and financial responsibilities may further increase maternal vulnerability, which may be exacerbated by climate change through increasing drought frequency and severity [12].

The adverse impact of perinatal depression on child health is well documented. Antenatally, it contributes to pregnancy loss, stillbirth, or preterm births [15, 16]. Postnatally, it contributes to infant malnutrition and febrile illness [16] and later, to developmental and behavioural problems [17]. Perinatal suicidal ideation affects about 2–5% of mothers but may rise to about 14% in deprived settings [18]. Although rare, completed perinatal suicide remains a significant public health concern. Infanticide may occur at a similar rate and warrants equal attention. Traditionally linked to psychosis or prior psychiatric admissions [19], infanticide is increasingly recognised as potentially arising from acute situational overwhelm or “blind rage” rooted in psychosocial stressors [20].

Most research in Kenya has focused on the mothers’ perspectives. Some studies have explored the views of health-care workers (HCWs) [21, 22] but few, if any, combine both. A dual perspective is needed to develop services not only effective in ideal conditions but also in real-world settings. The persistently high prevalence of perinatal depression alongside two highly publicised reports of maternal–child homicides that occurred in Kajiado County within three months of each other in 2023 [23, 24], underscores an urgent need to address maternal mental health and social determinants.

The aim of the study was to explore mental health problems, social needs, and associated risk factors in women ante- and postnatally in Kajiado County in Kenya from the perspectives of women and HCWs. The work represents the first step in a two-phased project to develop a context-based intervention package. The project works towards framework of the United Nations sustainable development goals (SDGs), a framework to address global development priorities. Specifically, our project will address three SDGs: 3: Good health and well-being, 5: Gender Equality, and 17: Partnerships for the goals [25].

## Methods

### Study design and participants

We conducted a qualitative study based on dual-perspective focus group interviews with mothers (ante- and postnatal) and HCWs in Kajiado County, Kenya between 5 and 11 March 2025. Kajiado County has a recorded population of about 1.1 million, bordering the capital, Nairobi, to the north and Tanzania to the south. The county includes both rapidly urbanising centres with an expanding multiethnic representation, as well as rural areas with a predominantly Maasai community. Pastoralism is common in parts of the rural areas [26, 27]. The geographic social and cultural diversity make Kajiado County a relevant model for communities in transition in low-income and middle-income countries.

**Fig. 1:**
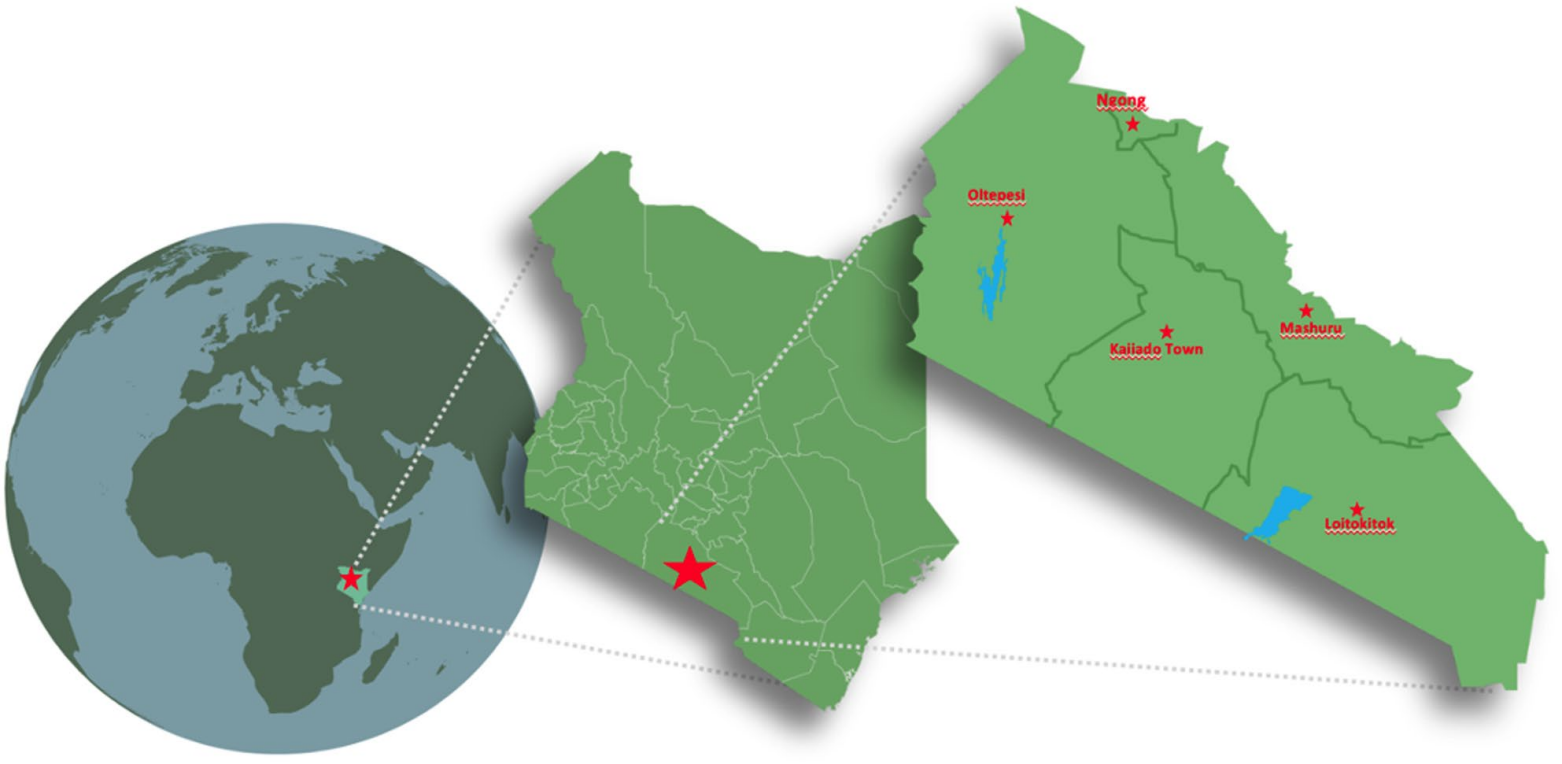
Location of study sites in Kajiado County

To capture participants with relevant experiences, while ensuring a diversity of perspectives, we used a purposive sampling strategy [28], recruiting mothers and HCWs in five healthcare facilities across Kajiado County, including rural and urban areas (Fig. 1).

All healthcare facilities were classified as level 3, or 4 or 5 with varying degrees of service provision. One healthcare facility was classified level 3, three level 4, and one level 5.

The research team comprised researchers from Kenya and Sweden, working together in a municipal partnership programme, with expertise in health, public health, and public mental health (Table 1).

**Table 1:** Focus group sites

Location	Health care facility	Facility level	Location
Kajiado East	Mashuru Sub County Hospital	4	Rural
Kajiado Central	Kajiado County Referral Hospital	5	Urban
Kajiado South	Loitoktok Sub County Hospital	4	Rural
Kajiado South	Ngong Sub County Hospital	4	Urban
Kajiado West	Oltepesi Health Centre	3	Rural

Level 3: Health centres without a doctor on site

Level 4: Subcounty hospitals with an operation theatre and a few specialist doctors

Level 5: County referral hospitals

Mothers were eligible to participate when they were at least 18 years old and were either pregnant or had given birth within the last year. HCWs were eligible if they were regularly involved with maternal health issues, either as direct care providers or support staff. We determined the sample size pragmatically, based on previously conducted studies, on-site resources, and time constraints. We considered it feasible and sufficient for data saturation, to recruit about ten mothers and eight HCWs per site resulting in a total sample of 40 HCWs and 50 mothers.

On the day of the interview, we approached women and HCW present at the respective facility and invited them to participate. Written informed consent was then obtained from all participants. Consent forms were provided in English and HCWs gave consent in English. Mothers who did not speak English were informed verbally in their own languages based on the consent form – including Maa, Swahili, Kamba– before providing written consent. Mothers who were illiterate provided consent via thumbprint.

The study was approved by the Institutional Scientific and Ethics Review Committee of Mount Kenya University (MKU/ISERC/3353MKU). A license to conduct the study was obtained from the National Commission for Science, Technology and Innovation (NACOSTI/P/25/415186). Authorisation to collect the data was obtained from the department of health in Kajiado County. Ethical approval was also obtained from the Swedish Ethical Review Authority (DNR 2024-00376-01). The study was conducted in accordance with the ethical principles outlined in the Declaration of Helsinki.

### Procedures

At each site, focus groups for mothers and HCWs were held separately to ensure that the perspectives of each group were captured without influencing one another. Focus groups with HCWs were conducted in English; those with mothers in local languages or English. Each focus group was led by a principal facilitator, a role undertaken by HCWs attached to the units where the interviews were conducted. HCW attached to the units were chosen because we judged that this might increase trust in the respondents and improve willingness to participate. All HCW facilitators received prior briefings from members of the research team. In addition, research team members were present as assistant facilitators.

The focus group discussions followed semi-structured interview guides developed by the research team (Appendix). Recordings were then transcribed verbatim and anonymised. Where applicable, transcripts were translated into English. They were then uploaded to a cloud-based data analysis programme for coding.

### Data analysis

We conducted a qualitative content analysis as described by Graneheim and Lundman 2004 [29]. Qualitative content analysis was considered appropriate because it emphasises the systematic interpretation and abstraction of both manifest and latent content through coding, categorisation and theme development. Given the exploratory nature of the study, this approach facilitated a structured examination of participants’ experiences while remaining grounded in the data. As the aim of the study was exploratory, the focus was on understanding experiences and meanings rather than quantifying responses [30].

We adopted an explorative and inductive approach, appropriate for an under-studied area, to develop themes to inform a subsequent large quantitative survey. The text was divided into meaning units and manually coded in Atlas.ti (https://atlasti.com). Themes, subthemes and categories were articulated following a stepwise, iterative and reflexive process of abstraction and interpretation. For manuscript preparation, quotations were lightly edited for spelling and grammar to improve readability without altering their meaning or tone.

### Trustworthiness

To ensure trustworthiness, members of the research group were present at all focus groups and engaged with the data from the point of collection. To harmonise the coding process, Kenyan and Swedish researchers jointly coded the first HCWs interview. The Kenyan team then led the coding of the mothers’ interviews to maximise understanding of culturally and contextually specific perspectives. The Swedish team led the coding of the HCWs interviews. During the data analysis, the full research team met online repeatedly to reflect upon and discuss the developing themes.

Throughout, we followed the Standards for Reporting Qualitative Research (SRQR) checklist (Appendix).

## Results

Of 69 women approached, 71.0% completed the full interview and 2.8% part of it. Of these, 62.7% were interviewed in a rural and 37.3% in an urban facility. Of 63 HCWs approached, 61.9% completed the full interview and 17.4% part of it. Of these, 56.0% were interviewed in a rural and 44.0% in an urban facility. No participant leaving the focus group early withdrew consent. About half of the mothers were aged 18–24 years and nearly two thirds identified as Maasai. Overall, 54.9% were pregnant and 45.1% had delivered within the preceding year. Among the HCWs, 40.0% were nurses, clinical officers or medical assistants and 10.0% were doctors. The remaining HCWs included a variety of professions allied to medicine, such as human immunodeficiency virus (HIV) testing service councillors, nutritionists and health-care information officers. The mean interview duration was 66.0 min (SD 15.4) for mothers and 66.8 min (SD 11.3) for HCW (Table 2).

**Table 2:** Participants characteristics

Mothers (*n* = 51)		Healthcare workers (*n* = 50)	
N completing the whole interview	49 (96.1%)		39 (78.0%)
N completing part of the interview	2 (3.9%)		11 (22.0%)
Duration of interview, min Mean (SD)	66.0 (15.4)		66.8 (11.3)
Languages used	Maasai Swahili English		Swahili English
Age, years			
18–24	25 (49.0%)		5 (10%)
25–30	14 (27.5%)		11 (22%)
31–35	2 (3.9%)		13 (26%)
36–40	4 (7.8%)		9 (18%)
> 40	1 (2.0%)		12 (24%)
Missing	5 (9.8%)		0 (0%)
Maternity status		Sex	
Pregnant	28 (54.9%)	Male	15 (30%)
Baby within the last year	23 (45.1%)	Female	33 (66%)
Missing	0 (0%)	Missing	2 (4%)
Ethnicity		Profession	
Maasai	33 (64.7%)	Nurse	11 (22%)
Luo	4 (7.8%)	Clinical officer, medical assistant	9 (18%)
Luhya	5 (9.8%)	Doctor	4 (8%)
Kikuyu	5 (9.8%)	Consultant	1 (2%)
Mixed/ other	4 (7.8%)	Other^a^	25 (50%)
Missing	0 (0%)	Missing	0 (0%)

^a^Community health assistant, community health promoter, health records and information officer, HIV testing services councillor, hospital administration officer, intern, medical laboratory technician, link desk officer, medical social worker, nutritionist, adherence counsellor, pharmacy, sonographer, physiotherapist

Three themes were developed across both groups (a) women’s autonomy and self-determination, (b) responsive maternal and mental health care, and (c) community knowledge and acceptance of mental health problems (Table 3).

**Table 3:** Themes, subthemes and categories describing women’s and health care workers perspectives on the mental health problems and social needs of women before and after childbirth in Kajiado County

Women	HCW	Women	HCW	Women	HCW
Themes					
Women’s autonomy and self-determination		Responsive maternal and mental health care		Community knowledge and acceptance of mental health problems	
Subthemes					
Support of family and friends		Accessibility and competence		Challenges in the community	
Categories					
Receiving support from family and friends and having someone to talk to	Being alone and lacking a support system	Not knowing where to get help for health issues	Limited access to care due to long distances and organization of care	Lack of understanding of mental health	Community dialogue involving local leaders works
Conflicts with family	Mothers and mothers in law become caretakers	Not attending clinics because of the distance	Negative outcomes because of mothers coming in late	Confiding in religious and spiritual leaders in matters of mental health	Men need to be involved in maternal health
Being lonely, lacking support from family and friends, and not being able to talk to them about mental health		Not being able to confide in male health care workers	Integrated services and HCWs reaching out can increase accessibility	Mental health is difficult to talk about	Women are not allowed to speak out
Lack of support from the child’s father		Not being able to pay for health care	HCW experience distress but are expected to act strong	Women have no voice in the community	Religious leaders are perceived as trustworthy but might delay health care
Arguing, abuse and not being able to meet the husband’s expectations		Waiting for another reason than mental health to come to hospital	HCW need support and training		Mental health needs political support
Having a husband who cares		Not seeking help because knowing that one will not get it	HCW lack time and resources to talk about mental health		Access to peer support
The husband being the one making the decisions		Having mental health professionals at health facilities makes mental health care accessible	HCW have the ability to recognise, assess and diagnose mental health problems		
Being abandoned by the child’s father			Mental health care needs to be pro-active		
Being pregnant causing stress and negative feelings			Follow up is a challenge		
Subthemes					
Material resources		Fear and uncertainty		Beliefs about mental health	
Categories					
Having to do household chores and care for animals when looking after children and being pregnant	Being burdened and bound by household chores	Traumatic birth experiences	Losing the baby is traumatic but there is not always time for follow up	Believing that mental health problems is being irrational, having a mental disorder, to ruminate or being possessed	Religion and spiritual beliefs explain mental illness
Being poor and lacking control over financial decisions	Socioeconomic pressure can make women feel they want to kill the child	Fear of caesarean section, miscarriage, and other complications	Birth related trauma and pain can cause distress	Believing that the cause of mental health problems is life events and lack of resources and assistance	Mental health stigma is real but can be reduced
Lack of proper and sufficient food	Poverty means difficulties paying for and accessing healthcare	Fear and uncertainty about giving birth Difficulties trusting and confiding in HCW	Babies might get infected with HIV which is unnecessary and traumatising	Believing mental health challenges should be kept a secret	
	Lack of food is a challenge		Mothers with mental health problems might neglect the baby		
	Men make the decisions; women’s work is to give birth		Mothers might fear caesarean section, miscarriage and having a disabled child because of stigma		
			Mothers need to be able to confide in HCW but sometimes distrust them		
Subthemes					
Gender-oppressive practices					
Categories					
Being forced to a marriage without love	Being subjected to gender-based violence				
Lack of safe abortion options	Children being forced to marriage				
Getting pregnant too young, out of wedlock, or unplanned	Young girls not being mentally or physically prepared for pregnancy and childbirth				
Not being in control of one’s reproductive health	Women do family planning in secret as men seek to control them				
Not giving birth to boys	Women and men need to be educated on family planning				
Being blamed for infertility	A preference for male children				
	Female genital mutilation causing trauma				
	Social exclusion due to unplanned pregnancy				
	Women being blamed for infertility				
	Fear of disclosure of HIV status				
Subthemes					
Education and employment					
Categories					
Pregnancy can mean losing your income or dropping out of school					
Having an income reduces stress and increases independence, but still having the burden of domestic chores					
Education is important for girls, but getting educated is not always possible or supported by parents					

*HCW *health care worker, *HIV *Human immunodeficiency virus

### Women’s autonomy and self-determination

Within this theme, mothers and HCW talked about how issues related to support of family and friends, material resources, and gender-oppressive practices, affected women’s mental health. Additionally, women also put forth issues of education and employment.

Mothers described themselves, and were perceived by HCWs, as stressed and distressed when having to manage pressures of being isolated, unsupported and lacking control over resources or decisions-making, while still shouldering the bulk of household responsibilities. One mother summarised this as follows: *“I want to do all these things in a day*,* which is very stressful”*. The combined burden of household responsibilities and childcare accumulating over the course of the day was described as affecting mood: *“Sometimes*,* let’s say from morning*,* when you wake up*,* you are good*,* your moods are okay in the morning hours. Now by midday*,* you start feeling tired*,* you feel dizzy*,* your body is just tired*,* and you have done nothing…”*. Mothers also expressed that their needs were ignored: *“you find that the husband loves the goats more than the mother and the children”* and that having someone who would listen or care would be helpful.

HCWs noted that socio-economic pressure could provoke extreme distress, especially when births were closely spaced: *“Maybe she had a delivery. 2024*,* 2025*,* she’s pregnant again. Spacing. You see this a big challenge. You find that times she has seven children*,* eight*,* not employed*,* no farm*,* the drought. So*,* when she comes to maternity*,* or during delivery*,* most of the time you find at times even they want to kill the baby*”.

Negative consequences on mental health from gender-oppressive practices were evident in accounts of forced (arranged) marriage, coerced reproduction, and intimate partner violence (IPV): One woman described: “*if he wants you to have another child and you have no choice*,* he will force you”.* IPV was sometimes presented as conditional: “*if you agree to give birth you won’t be beaten*”. HCWs further highlighted the vulnerability of girls subjected to early marriage and female genital mutilation (FGM), often unprepared for motherhood: *“the girl is given out after the FGM*,* and this girl is not mentally prepared. She has not prepared to get married. …. Then later at night at about 2 am*,* she was woken up. The next thing she found herself is that she was in another home”.* Such unpreparedness and lack of involvement in decision-making were described as associated with depressive symptoms: “*So there is that marriage where you are not prepared*,* the forced marriage or the early marriage. And these girls*,* usually to post-traumatic depression*,* you find that they are quite depressed because it is not from their own. It is something which has been planned out for them.”*

HCW described FGM conducted in young girls against their will as “*mental torture*” and as both mentally and physically traumatic: *“…that is where the trauma starts*,* the pain you go through*,* they hold you*,* they cut*,* and now the healing process”* At the same time, a traditional view that a women was not complete without FGM and a need for social inclusion would make a girl wanting FGM, although this was becoming less prevalent.

Both women and HCWs observed that women were blamed for infertility or not giving birth to boys. Mothers also described how pregnancy could result in the loss of education and independent income, further eroding autonomy.

HIV was not raised by women but only emerged in the interviews with the HCW as a stigmatising and emotionally conflicting condition associated with guilt and contributing to depression. Women’s HIV test result could also disclose the husband’s status. Yet, women might choose not to disclose their HIV status to their husbands, might not adhere to treatment and in some cases withhold medications from their infants, leading to fear of infecting the baby. One HCW described this as follows: *“So for them*,* they found it very difficult because we have now the fear of infecting the baby. Also*,* the fear of the unknown. How will my partner or family react? If they find out that I am positive…”*.

### Responsive maternal and mental health care

In this theme, women and HCWs alike described issues of *accessibility and competence* of HCW, and women’s experiences of *fear and uncertainty* as relevant to maternal mental health. Women described structural and psychological barriers to accessing care, including distance, cost, low expectations of receiving help, lack of knowledge of how to seek help, and expectations to continue household responsibilities close to childbirth. In a rural setting, women described giving birth alone using a razor and a rope: “*they carry a small thread*,* in case of anything*,* if she has had a child*,* she will bring it home*…*you go with a goat*,* you carry a rope*,* the baby comes out*,* you cut the rope.”* Such experiences were described as causing anxiety related to childbirth. Despite this, women reported feeling compelled to have another child as soon as possible: *“…he has seen that you have given birth in a bush*,* he does not care*,* he is waiting for the child to be small and has not even reached a year*,* he wants you to have another child and you have no choice*,* he will force you…”*., which was described as contributing to psychological distress and affecting mental wellbeing: *“You will find that another is stressed*,* has started to go mad*,* another wants to commit suicide*,* things like that*”.

Some participants hesitated to seek help unless they had physical symptoms, which could serve as a pretext. In addition, HCWs identified distance as one barrier to access, which could contribute to delayed presentations. Delayed presentation for care was described as potentially worsen pregnancy outcomes, including adverse outcomes such as cerebral palsy. In such situations, mothers might sometimes hide the baby from view because of stigma or lack of knowledge instead of seeking help. HCW also stressed the need for service organisation and integration of mental and proactive mental health care but noted constraints in time and resources. Follow-up was particularly difficult. HCWs reported their own emotional strain but felt pressured to appear strong.

Women expressed deep fears about traumatic or painful births, miscarriage, and particularly caesarean section (CS): “*if you have CS*,* you will die …my mum was telling me CS is not good*,* it hurts a lot*,* so I was just praying to God not to have CS*”. HCWs described CS as often stigmatised, which could delay care-seeking and affect mental wellbeing: “*We usually get referrals for when things have gone so far…There is no sign of happiness on their face*,* unlike in communities. … So*,* you find that there’s a lot of stigma in the community if you deliver by CS…”* In addition, CS, particularly when resulting in a loss of the child, was also perceived as emotionally challenging by both mothers and HCW: “*the only memory of the child was maybe it was CS”*. FGM was cited as a complicating factor: *So*,* we go to theatre and you find that this woman has tried out there to give up the FGM was done and you can clearly see there’s so much obstruction scar tissue around that.”*

Both women and HCW raised concerns about lack of trust and the difficulty of confiding in HCWs. There were concerns about negative consequences of confiding in HCW, particularly when they were male, since they might share information with the husband: *“…and the doctor is a friend of the husband*,* he called the husband….”*. Some participants expressed distrust that HCW might make mistakes and thereby compromise birth outcomes. In one rural site, women were particularly concerned about students conducting medical procedures: “ *People are very afraid of students”*,* “I am not sure if I am told that students are the ones who are destroying people*” and did not feel that they could decline care delivered by students.

Feeling insufficiently informed and involved in their care was described as increasing anxiety and potentially contributing to depression. Shame prevented women from talking about mental health problems. HCW emphasised the importance of confidentiality to enable women to speak openly without fear of exposing themselves or their community. Crowded clinics, however, could compromise confidentiality. Dealing with a grieving mother after child loss was seen as particularly difficult and emotionally distressing.

### Community knowledge and acceptance of mental health problems

In this theme, both women and HCWs talked about how *challenges in the community* and *beliefs about mental health* affected mental health during pregnancy and child-birth. Women described how mental health problems were widely viewed as irrationality or spiritual possession, leading women to confide in religious or spiritual leaders. Mental health remained a taboo topic that could not be raised with men: “*They do say that women have no voice in the community… You can’t complain*,* that is like you say if you’re a woman it’s not okay*,* that is you can’t complain in front of men*”. Some women could confide in their mothers and sisters though. Women generally reported that they would be willing to discuss their mental health with HCWs; however, they perceived that there were limited resources for mental health care and that patients with physical conditions would be prioritised.

HCWs echoed this view noting that physical problems and immunisations were prioritised and that resources for mental health care were insufficient. One HCW further described mental health as stigmatised – comparable to HIV – which could limit open discussion and help-seeking: *“I think the reason why mental health is silent is a stigma. Just the way HIV was introduced with a very stigmatizing scandal. Mental health has been stigmatised to the true psychotics*,* the ones who are walking around naked*,* screaming and everything.”* HCWs described how religious and spiritual explanation could delay mental health care: “*Number one*,* you find these mothers are taken to spiritual leaders. These mothers are taken for these traditional healer and the time they come to the hospital*,* it’s unmanageable.*” They emphasised the importance of engaging religious leaders and shifting community perceptions: “*as healthcare workers*,* it is our role to advise the spiritual leaders that sometimes these conditions are not spiritual or they’re not demonic*,* but they’re mental issues.*” Improving mental health acceptance was seen to need community dialogue with local leaders, male involvement, and peer support to empower women.

## Discussion

This study is the first to explore maternal mental health in Kajiado County and reveals how closely it is tied to entrenched gender norms and prevailing perceptions of mental health. Our dual-perspective approach – exploring views from both mothers and HCWs – offers a deeper understanding by highlighting the social and emotional stressors and stigma faced by women while also pointing to systemic barriers, provider distress, as well as mismatched expectations and priorities. Additionally, our findings highlight maternal mental health challenges related to urban–rural transition and pastoralist livelihoods, which appear to be less commonly addressed in perinatal research. Although rooted in Kajiado County, our findings may be relevant to other low-income and middle-income settings undergoing similar transition with urbanisation, shifting living conditions, and evolving gender dynamics.

Our findings resonate with a systematic review and meta-analysis of 42 studies on postnatal depression in sub-Saharan Africa, in which factors associated with postnatal depression were categorised into three levels based on their relative importance [9]. Level-one factors included IPV or conflict, poor social support, unplanned pregnancy, and young maternal age. Level-two factors included maternal illness, single motherhood, poor socioeconomic status, and low education. Level-three factors comprised perinatal death, undesired infant sex, CS, substance abuse, antenatal depression, poor infant health, and primiparity. These factors reflect our first two themes, “Women’s autonomy and self-determination” and “Responsive maternal and mental health care” and highlight women’s psychological, physical, and social vulnerabilities as risk factors for both antenatal and postnatal depression [5, 8]. However, our third theme, “Community knowledge and acceptance of mental health problems”, while touched on in the aforementioned review, was not explicitly described as a distinct factor. In this regard, our findings resonate with an international expert review, which described sociocultural and family environments as maintaining factors – “a gulf between how women feel and a web of norms and expectations surrounding motherhood” – and highlighted stigma and low mental health literacy as major barriers to care [1]. If mental health issues are not addressed, the risk of perinatal depression may increase, highlighting the need for proactive interventions [31]. Our findings suggest that the community perspective is important and merits incorporation in further research and practice development in this area.

The wide range of contributing factors, however, can make it challenging to formulate meaningful and affordable solutions. Many studies acknowledge this complexity of the problem but fall short of suggesting concrete feasible interventions for resource-constrained settings. While health- care interventions often address the consequences of underlying problems, community interventions can target root causes. For example, Nweke et al. 2022 recommend targeted screening, psychotherapy, conflict resolution, and social support including child-focused programmes [9]. Kariuki et al. 2021 demonstrated that a brief, one-off, psychoeducational intervention could significantly reduce postnatal depression by 36% among mothers living in Nairobi informal settlements within six months although the long-term benefits remain unclear [32]. Without addressing community-level barriers and empowering women to access care, such measures may have limited long-term impact. One systematic review identified lack of confidentiality, judgemental attitudes among HCWs, and poorly tailored services as key barriers to effective mental health support for pregnant adolescents and young women. It recommended integrating mental health services into school, community, and dedicated health-care settings [33].

More resources for the health-care system alone are unlikely to lead to better results. The three themes described in our qualitative analysis suggest that health-care interventions may benefit from being integrated with community-based approaches to achieve sustainable improvements in maternal mental health. Reduction of gender-oppressive practices calls for tackling forced marriage, coerced reproduction, and IPV. However, women’s empowerment may be insufficient, unless the underlying restrictive gender norms are also challenged [34]. This would likely involve proactive – and culturally sensitive – male partner participation (MPP), which remains low in Kenya and other countries sub-Saharan Africa with reported prevalence figures ranging 18% to 32% [35]. The role of churches and traditional spiritual practices also warrants further investigation. Although they may conceptualise mental health differently, both exert substantial influence on health-seeking behaviour.

Our results suggest that fear of pain, CS, and poor birth outcomes may contribute to delayed care in contexts where antenatal services are widely perceived by women as stigmatising and unsafe. In interpreting the findings, it is important to note that they reflect differing perspectives, and that some views expressed by HCWs were not explicitly present in women’s narratives.

While women expressed fear of CS, they did not describe the link between delayed antenatal care and the need for CS in their accounts. FGM was not mentioned by women but raised by HCWs, who discussed its potential relevance to obstructed labour. HCWs also described experiencing stress in the context of obstetric emergencies and adverse outcomes.

Proactive education at both individual and community levels may support maternal and HCW wellbeing. At the same time, addressing FGM remains important. Previous research has reported as association between FGM and an increased risk of CS in primiparous women (1.8-fold) and postpartum haemorrhage (2.6-fold) in African settings [36]. Addressing FGM requires open community dialogue, which may be difficult where the practice is culturally ingrained. A pragmatic strategy may involve education about the link between FGM and adverse birth outcomes, emphasising that improving child health depends on protecting maternal health: healthy mothers mean healthy children. Distance to the healthcare facilities was identified as a major barrier delaying essential care. In one rural case, a patient had walked overnight to reach antenatal services. Mobile clinics could help improve access in such settings.

One limitation of this study was the heterogeneity in the composition of HCWs participating in the focus groups discussions, which varied by site. This was primarily due to practical constraints, including differences in staff availability, such as a nurses’ strike at one facility on the day of the discussion. However, this heterogeneity also reflects the staffing realities in these settings, where limited human resources require HCWs to take on multiple roles. Another limitation was the absence of male participants. The male perspective is vital to understanding barriers to and opportunities for change. Prioritisation of livestock may reflect an economic necessity or cultural status. Yet– as our findings show – it could also cause emotional distress in women. The qualitative nature of this research inherently limits generalisability, but the findings serve as a valuable foundation for quantitative research. Finally, HIV/ acquired immunodeficiency syndrome (HIV/ AIDS) – a condition often associated with gender-oppressive practices – did not emerge prominently in the interviews with women. This may reflect the highly stigmatising and emotionally conflicting nature of HIV as indicated in interviews with HCWs, which may have limited women’s willingness to discuss HIV and facilitators’ ability to ask probing questions.

A further limitation relates to the use of HCWs attached to the units where the interviews were conducted as principal facilitators. This approach may have increased the risk of interviewer-respondent bias, particularly if respondents felt pressure to participate or to respond in certain ways due to concerns about their clinical care (in the case of mothers) or professional standing (in the case of the HCW). However, it was made explicit during the consent procedures that participation was entirely voluntary and that participants could withdraw at any time. Methods to mitigate this risk included prior briefing of HCW facilitators and the presence of research team members as assistant interview facilitators.

English translations may not always capture the original meaning. For instance, the Swahili word “kupenda” can mean both, “to love” and “to like”. To reduce misinterpretation, we used professional translators and discussed meanings extensively within our multi-lingual research team.

While the resulting themes may resemble those that could be generated through thematic analysis [37], qualitative content analysis was considered most appropriate because it emphasises the systematic interpretation and abstraction of both manifest and latent content through coding, categorisation and theme development. Given the exploratory nature of the study, this approach facilitated a structured examination of participants’ experiences while remaining grounded in the data [30]. Ultimately, as a qualitative study, our findings do not establish frequencies or statistical associations but instead identify factors and experiences that may influence perinatal mental health within this context, contributing to an enhanced understanding of the complexity of these experiences.

## Conclusion

Our findings describe how maternal mental health is closely intertwined with gender norms and prevailing perceptions of mental illness including stigma. Expanding maternal mental health services may be important but it is unlikely to be sufficient in isolation. Sustainable change may depend on the promotion of women’s rights, increased mental health literacy at the community level, and the engagement of men. In addition, fear of traumatic birth and stigma associated with CS may need to be addressed not only at the individual level but also within the broader community context.

## Supplementary Information

Supplementary Material 1.

## Data Availability

The datasets generated and/or analysed for the current study are not publicly available due to lack of ethics committee permission and not having been part of the consent process. Any reasonable request will be raised with the regional ethics committee and healthcare provider.

## Abbreviations

AIDS: Acquired immunodeficiency syndrome
CS: Caesarean section
COVID-19: Corona virus-19 disease
FGM: Female genital mutilation
HCW: Health-care worker
HIV: Human immunodeficiency virus
IPV: Intimate partner violence
MPP: Male partner participation
Min: Minutes
N: Number
PTSD: Post-traumatic stress disorder
SD: Standard deviation
SDGs: Sustainable development goals
SRQR: Standards for Reporting Qualitative Research
