# Supplementary material for: Exploring potential factors influencing perinatal mental health in Kajiado County, Kenya: a qualitative study with mothers and healthcare workers
Source: BMC Public Health. 2026 Jun 23;26:1950. doi: 10.1186/s12889-026-28282-4 (PMC13292506; doi:10.1186/s12889-026-28282-4)
Supplement: Supplementary file 1 — Supplementary Material 1. [file 12889_2026_28282_MOESM1_ESM.docx]

**Appendix**

**Interview guides**

***Mothers’ focus groups***

What is your age range?

- 18–24 years
- 25–30 years
- 31– 35 years
- 36–40 years
- > 40 years

What is your maternity status?

- I am currently pregnant.
- I had a baby within the last year.

1. **Introductory**
   - How have you been feeling emotionally before and after childbirth?
   - What are the most significant challenges you've faced in terms of mental well-being during this period?
   - Can you describe a typical day and its impact on your mental health during pregnancy or post-childbirth?
2. **Perceptions and Needs**
   - What do you believe are the key mental health concerns for women before and after childbirth in this community?
   - What kind of support, if any, have you received or wished for concerning mental health during this phase?
   - How comfortable are you discussing mental health issues with healthcare workers?
   - What do you think are the causes of mental health issues?
   - What does your family think are the causes of mental health issues?
   - What do you think the community believes are the causes of mental health issues?
   - Is anybody to blame for mental health issues? And why are they to be blamed?
3. **Access and Barriers**
   - Have you encountered any obstacles in accessing mental health services before or after childbirth?
   - What changes would make it easier for women in this community to access mental health support during these periods?
4. **Community and Social Support**
   - How does your community respond to or assist women facing mental health challenges during pregnancy or after childbirth?
   - Have cultural practices or beliefs influenced your mental health journey during this time?
   - What are the likely causes of mental health challenges during and after child birth?
   - Which are the root causes that can be addressed and which of the causes that cannot be addressed ?
   - What would make things better?
   - What would make things worse?
   - Is it acceptable to talk about mental health in the household, community and at the health facility?

***Healthcare workers’ (HCW) focus groups***

What is your age range?

- 18–24 years
- 25–30 years
- 31– 35 years
- 36–40 years
- 41–55 years
- >55 years

What is your sex?

- Male
- Female
- Do not want to say

What is your profession?

- Nurse
- Clinical officer /Medical assistance
- Doctor
- Consultant
- Other, please specify

1. **Understanding Screening and Identification:**
   - What are the key challenges you face in identifying mental health issues in pregnant or postpartum women during routine care?
   - Can you share instances in the last 2 years where social factors significantly influenced a woman's mental health during or after pregnancy?
   - How equipped do you feel in recognizing severe mental disorders like bipolar disorder or severe depression, psychosis in these women?
2. **Intervention Development:**
   - From your experience, what specific interventions other than medication do you believe could effectively address mental health issues in this demographic?
   - How important do you think it is for interventions to not only treat symptoms but also tackle the root causes of these mental health challenges?
   - In your opinion, what role should community health care workers play in delivering mental health support to these women?
3. **Impact on Child Health:**
   - Based on your observations, how do you see a mother’s mental health impacting the well-being of her child during the first year of life?
   - Have you encountered situations where a mother's pre-existing mental illness affected the child’s health? If so, how?

**SRQR checklist**

|  | Standards for Reporting Qualitative Research (SRQR)* | MANUSCRIPT 1: QUALITATIVE ANALYSIS |
| --- | --- | --- |
|  | <http://www.equator-network.org/reporting-guidelines/srqr/> |  |
|  |  | Page/line no(s). |
| Title and abstract | |  |
|  | Title - Concise description of the nature and topic of the study Identifying the study as qualitative or indicating the approach (e.g., ethnography, grounded theory) or data collection methods (e.g., interview, focus group) is recommended | Title:  Exploring potential factors influencing perinatal mental health in Kajiado County, Kenya: A qualitative study with mothers and healthcare workers |
|  | Abstract - Summary of key elements of the study using the abstract format of the intended publication; typically includes background, purpose, methods, results, and conclusions | Included |
|  |  |  |
| Introduction | |  |
|  | Problem formulation - Description and significance of the problem/phenomenon studied; review of relevant theory and empirical work; problem statement | Included with concluding problem statement: “The persistently high prevalence of perinatal depression alongside two highly publicised reports of maternal–child homicides that occurred in Kajiado County within three months of each other in 2023 [23,24] underscores an urgent need to address maternal mental health and social determinants.” |
|  | Purpose or research question - Purpose of the study and specific objectives or questions | Included: “The aim of the study was to explore mental health problems, social needs, and associated risk factors in women pre- and postnatally in Kajiado County in Kenya from the perspectives of women and HCWs. The work represents the first step in a three-phase mixed-method project to develop a context-based intervention package. The project works towards framework of the United Nations sustainable development goals (SDGs), a framework to address global development priorities. Specifically, our project will address three SDGs: 3: Good health and well-being, 5: Gender Equality, and 17: Partnerships for the goals [25]. |
| Methods | |  |
|  | Qualitative approach and research paradigm - Qualitative approach (e.g., ethnography, grounded theory, case study, phenomenology, narrative research) and guiding theory if appropriate; identifying the research paradigm (e.g., postpositivist, constructivist/ interpretivist) is also recommended; rationale** | Included:  --Dual-perspective focus group-based interview study in mothers (pre- and postnatal) and health-care workers in Kajiado County, Kenya  -- Purposive sampling strategy,^20^ recruiting mothers and HCWs in five healthcare facilities across Kajiado County, including rural, periurban, and urban areas (appendix)  --Qualitative content analysis as described by Graneheim and Lundman 2004.^21^ We chose an explorative and inductive approach, appropriate for an under-studied area, to develop themes to inform a subsequent large quantitative survey. |
|  | Researcher characteristics and reflexivity - Researchers’ characteristics that may influence the research, including personal attributes, qualifications/experience, relationship with participants, assumptions, and/or presuppositions; potential or actual interaction between researchers’ characteristics and the research questions, approach, methods, results, and/or transferability | Included:  “The research team comprised researchers from Kenya and Sweden, working together in a municipal partnership programme, with expertise in health, public health, and public mental health.”  “Each focus group was led by a principal facilitator, a role undertaken by HCWs attached to the units where the interviews were conducted. HCW attached to the units were chosen because we judged that this might increase trust in the respondents and improve willingness to participate. All HCW facilitators received prior briefings from members of the research team. I addition, research team members were present as assistant facilitators.” |
|  | Context - Setting/site and salient contextual factors; rationale** | Included: “Kajiado County has a recorded population of about 1.1 million, bordering the capital, Nairobi, to the north and Tanzania to the south. The county includes both rapidly urbanising centres with an expanding multiethnic representation, as well as rural areas with a predominantly Maasai community. Pastoralism is common in parts of the rural areas [26,27]. The geographic social and cultural diversity make Kajiado County a relevant model for communities in transition in low-income and middle-income countries.” |
|  | Sampling strategy - How and why research participants, documents, or events were selected; criteria for deciding when no further sampling was necessary (e.g., sampling saturation); rationale** | To capture participants with relevant experiences, while ensuring a diversity of perspectives, we used a purposive sampling strategy [28], recruiting mothers and HCWs in five healthcare facilities across Kajiado County, including rural and urban areas (Figure 1).  **Figure 1: Location of study sites in Kajiado County.**  All healthcare facilities were classified as level 3, or 4 or 5 with varying degrees of service provision. One healthcare facility was classified level 3, three level 4, and one level 5. |
|  | Ethical issues pertaining to human subjects - Documentation of approval by an appropriate ethics review board and participant consent, or explanation for lack thereof; other confidentiality and data security issues | Included: “Written informed consent was then obtained from all participants. Consent forms were provided in English and HCWs gave consent in English. Mothers who did not speak English were informed verbally in their own languages based on the consent form – including Maa, Swahili, Kamba– before providing written consent. Mothers who were illiterate provided consent via thumbprint.”  “The study was approved by the Institutional Scientific and Ethics review Committee of Mount Kenya University (MKU/ISERC/3353MKU). A license to conduct the study was obtained from the National Commission for Science, Technology and Innovation (NACOSTI/P/25/415186). Authorisation to collect the data was obtained from the department of health in Kajiado County. Ethical approval was also obtained from the Swedish Ethical Review Authority (DNR 2024-00376-01). The study was conducted in accordance with the ethical principles outlined in the Declaration of Helsinki.” |
|  | Data collection methods - Types of data collected; details of data collection procedures including (as appropriate) start and stop dates of data collection and analysis, iterative process, triangulation of sources/methods, and modification of procedures in response to evolving study findings; rationale** | Included: “We conducted a dual-perspective focus group-based interview study in mothers (pre- and postnatal) and health-care workers in Kajiado County, Kenya between 5 and 11 March 2025.”  “At each site, focus groups for mothers and HCWs were held separately to ensure that the perspectives of each group were captured without influencing one another. Focus groups with HCWs were conducted in English; those with mothers in local languages or English. Each focus group was led by a principal facilitator, a role undertaken by HCWs attached to the units where the interviews were conducted. HCW attached to the units were chosen because we judged that this might increase trust in the respondents and improve willingness to participate. All HCW facilitators received prior briefings from members of the research team. In addition, research team members were present as assistant facilitators.“ |
|  | Data collection instruments and technologies - Description of instruments (e.g., interview guides, questionnaires) and devices (e.g., audio recorders) used for data collection; if/how the instrument(s) changed over the course of the study | Included: The focus group discussions followed semi-structured interview guides developed by the research team (Appendix).” |
|  | Units of study - Number and relevant characteristics of participants, documents, or events included in the study; level of participation (could be reported in results) | Included: “We determined the sample size pragmatically, based on previously conducted studies, on-site resources, and time constraints. We considered it feasible and sufficient for data saturation, to recruit about ten mothers and eight HCWs per site resulting in a total sample of 40 HCWs and 50 mothers. “  Table 2 |
|  | Data processing - Methods for processing data prior to and during analysis, including transcription, data entry, data management and security, verification of data integrity, data coding, and anonymization/de-identification of excerpts | Included: “Recordings were then transcribed verbatim and anonymised. Where applicable, transcripts were translated into English. They were then uploaded to a cloud-based data analysis programme for coding.” |
|  | Data analysis - Process by which inferences, themes, etc., were identified and developed, including the researchers involved in data analysis; usually references a specific paradigm or approach; rationale** | “We conducted a qualitative content analysis as described by Graneheim and Lundman 2004.^21^ We chose an explorative and inductive approach, appropriate for an under-studied area, to develop themes to inform a subsequent large quantitative survey. The text was divided into meaning units and manually coded in Atlas.ti (https://atlasti.com). Overarching themes were articulated following a stepwise, iterative and reflexive process of abstraction and interpretation involving the creation of domains, categories and sub-themes. For manuscript preparation, quotations were lightly edited for spelling and grammar to improve readability without altering their meaning or tone.” |
|  | Techniques to enhance trustworthiness - Techniques to enhance trustworthiness and credibility of data analysis (e.g., member checking, audit trail, triangulation); rationale** | Included: “To ensure trustworthiness, members of the research group were present at all focus groups and engaged with the data from the point of collection. To harmonise the coding process, Kenyan and Swedish researchers jointly coded the first HCWs interview. The Kenyan team then led the coding of the mothers’ interviews to maximise understanding of culturally and contextually specific perspectives. The Swedish team led the coding of the HCWs interviews. During the data analysis, the full research team met online repeatedly to reflect upon and discuss the developing themes. “ |
|  |  |  |
| Results/findings | |  |
|  | Synthesis and interpretation - Main findings (e.g., interpretations, inferences, and themes); might include development of a theory or model, or integration with prior research or theory | Included in text and table 2 |
|  | Links to empirical data - Evidence (e.g., quotes, field notes, text excerpts, photographs) to substantiate analytic findings | Field quotes included in the text |

| Discussion | |  |
| --- | --- | --- |
|  | Integration with prior work, implications, transferability, and contribution(s) to the field - Short summary of main findings; explanation of how findings and conclusions connect to, support, elaborate on, or challenge conclusions of earlier scholarship; discussion of scope of application/generalizability; identification of unique contribution(s) to scholarship in a discipline or field | Included in the discussion. |
|  | Limitations - Trustworthiness and limitations of findings | Included in the discussion. |
|  |  |  |
| Other | |  |
|  | Conflicts of interest - Potential sources of influence or perceived influence on study conduct and conclusions; how these were managed | Included: All authors provided a declaration |
|  | Funding - Sources of funding and other support; role of funders in data collection, interpretation, and reporting | Included: Swedish International Centre for Local Democracy (ICLD) grant no 2024-2020/ 2024-2021 and Insamlingsstiftelsen Umeå University, Sweden, grant no1002287. |
|  |  |  |
|  | *The authors created the SRQR by searching the literature to identify guidelines, reporting standards, and critical appraisal criteria for qualitative research; reviewing the reference lists of retrieved sources; and contacting experts to gain feedback. The SRQR aims to improve the transparency of all aspects of qualitative research by providing clear standards for reporting qualitative research. |  |
|  |  |  |
|  | **The rationale should briefly discuss the justification for choosing that theory, approach, method, or technique rather than other options available, the assumptions and limitations implicit in those choices, and how those choices influence study conclusions and transferability. As appropriate, the rationale for several items might be discussed together. |  |
|  |  |  |
|  | Reference: |  |
|  | O'Brien BC, Harris IB, Beckman TJ, Reed DA, Cook DA. Standards for reporting qualitative research: a synthesis of recommendations. *Academic Medicine*, Vol. 89, No. 9 / Sept 2014  DOI: 10.1097/ACM.0000000000000388 |  |
|  |  |  |
|  |  |  |
